# High-testosterone men reject low ultimatum game offers

**DOI:** 10.1098/rspb.2007.0546

**Published:** 2007-07-05

**Authors:** Terence C Burnham

**Affiliations:** Program for Evolutionary Dynamics, Harvard UniversityOne Brattle Square, Suite 6, Cambridge, MA 02138, USA

**Keywords:** ultimatum game, behavioural economics, testosterone, reciprocal altruism

## Abstract

The ultimatum game is a simple negotiation with the interesting property that people frequently reject offers of ‘free’ money. These rejections contradict the standard view of economic rationality. This divergence between economic theory and human behaviour is important and has no broadly accepted cause. This study examines the relationship between ultimatum game rejections and testosterone. In a variety of species, testosterone is associated with male seeking dominance. If low ultimatum game offers are interpreted as challenges, then high-testosterone men may be more likely to reject such offers. In this experiment, men who reject low offers ($5 out of $40) have significantly higher testosterone levels than those who accept. In addition, high testosterone levels are associated with higher ultimatum game offers, but this second finding is not statistically significant.

## 1. Introduction

In the ultimatum game, one person (‘proposer’) makes an offer to a second person (‘responder’) on how to divide a sum of money. This offer is final—an ultimatum—so if the responder rejects it, there is no agreement, and neither person receives any money. Since rejections result in no money for either party, economic theories of self-interest predict that responders will accept all positive offers (Stahl 1972; Rubinstein 1982).

Contrary to these predictions, the first ultimatum game experiment reported that low offers were frequently rejected (Guth *et al.* 1982). This deviation between behaviour and that predicted by standard theory has been replicated in myriad studies (Roth 1995), including games played for large stakes (Hoffman *et al.* 1996; Cameron 1999) and cross-culturally (Roth *et al.* 1991; Henrich *et al.* 2001).

There is no broadly accepted explanation for these rejections, which contradict the standard definition of rationality. One prominent suggestion is that people have a taste for ‘fairness’, and thus ultimatum game rejections make people not only poorer, but also happier (Bolton 1991; Rabin 1993; Fehr & Schmidt 1999). If fairness is the proximate cause of ultimatum game rejections, it raises the question of the origin of this preferences. One suggestion is that people act ‘as if’ there is some chance that accepting low offers will damage their reputation and cost them in future interactions (Page & Nowak 2000). If it is possible that behaviour will be repeated (or simply observed), then the rejection of small offers may be rational (Alexander 1987, 2006; Bolton 1997; Ellingsen 1997; Nowak *et al.* 2000). This reputation-management machinery may be used by experimental subjects even in anonymous settings with no opportunity to develop reputations.

Since testosterone modulates behaviour across many species, and in settings that may be construed to be similar to the ultimatum game, it allows an interesting test of this explanation. If ultimatum game rejections result from the inappropriate activation of reputation-management machinery, then a body of research suggests that rejections will be more probable among high-testosterone men.

High-testosterone animals are more likely to respond aggressively to a challenge, and low offers may be viewed as challenges. Across multiple species, including humans, high testosterone levels are correlated with dominance-seeking behaviour (Mazur & Booth 1998), and dominants are less likely to back down from challenges.

There is considerable cross-species support for the role of testosterone in status and aggression. In a number of bird species, exogenous testosterone increases male–male competition (Silverin 1980; Hegner & Wingfield 1987). High-ranking chimpanzees (*Pan troglodytes*) have higher testosterone levels than low-ranking individuals, and they are more aggressive (Muller & Wrangham 2004). Similarly, dominant male wild mountain gorillas (*Gorilla gorilla beringei*) have higher testosterone levels than subordinate males (Robbins & Czekala 1997).

Among men, there is a consistent, positive relationship between aggression and testosterone (Book *et al.* 2001). High-testosterone men are rated as less friendly and more dominant (Dabbs 1997). Men who are looking for sexual partners, and therefore engaging in a form of male–male competition, have higher testosterone levels than those who are not seeking partners (Dabbs & Booth 1993; Burnham *et al.* 2003; Gray *et al.* 2004; McIntyre *et al.* 2006).

Adaptive models of aggression suggest that high-testosterone animals are more willing to incur the costs of conflict because of the compensating benefits (Mazur 1973, 1983, 1985; Wingfield 1984; Kemper 1990; Wingfield *et al.* 1990). In short, testosterone modulates a reputation-management system, where high-testosterone males are more willing to engage in conflict (Ellison 2001). In a review article on punishment, Clutton-Brock & Parker (1995) conclude that ‘negative reciprocity is used by dominant animals to resist subordinate members from indulging in a behaviour that threatens the fitness of the dominant members’.

Consistent with this view, a recent study reports that low second-to-fourth digit (2D : 4D) ratios are associated with men's willingness to reject low ultimatum game offers (Van den Bergh & Dewitte 2006). Low 2D : 4D ratios are believed to reflect high foetal exposure to androgens (Manning *et al.* 1998), but the relationship between 2D : 4D ratios and adult circulating T levels is ambiguous (Manning *et al.* 2004). One study reports that testosterone injections increase punishment levels among male subjects in an economic game (Kouri *et al.* 1995), which suggests that men with naturally high testosterone levels may reject low ultimatum game offers.

Do people interpret low ultimatum game offers as a challenge? In a neuroeconomic study, low ultimatum game offers caused increased brain activation in the anterior insula, a brain region associated with anger and disgust (Sanfey *et al.* 2003). Furthermore, subjects in this neuroeconomic study were more likely to reject low offers from people than similar offers from a computer, consistent with the reputation-management hypothesis and an earlier study (Blount 1995).

In summary, if men interpret low ultimatum game offers as a challenge, then those with higher testosterone levels will be more likely to reject the offer.

## 2. Material and methods

Thirty subjects began the experiment and 26 completed (four subjects were not present on the day of the ultimatum game). All subjects were male graduate students enrolled at Harvard University. Each subject had completed a minimum of two semesters of graduate microeconomics including an introduction to game theory. The experiment was approved by the Harvard University's committee on the use of human subjects in research.

The game was played for stakes of $40. Each pair was paid. All aspects of the game were public knowledge to all players, and there was no deception. Subjects were asked for their behaviour in both roles, proposer and responder, then paired anonymously. Roles were assigned randomly after all decisions were made. Subjects were paid based on their choices.

Ultimatum offers were constrained to be either $25 out of $40 or $5 out of $40. These two choices were picked to generate a roughly even split between high and low offers. Given that the focus of the experiment was on rejection behaviour, it was desirable to make the low offer a probable proposal. In many published studies, low offers are rare and median offers are close to half of the available money (e.g. Guth *et al.* 1982). Some studies that focus on rejections of low offers do not use real offers (e.g. Sanfey *et al.* 2003). The particulars of this experiment were designed to have subjects face a significant probability of a low offer actually given by the proposer.

All the testosterone assays were performed by the author. Saliva samples were identified only by an anonymous subject ID, so that testosterone levels were assessed without any knowledge of the subjects' ultimatum game behaviour. Testosterone was sampled from saliva using a methodology developed by Peter Ellison and members of his laboratory (Ellison 1988). Collection tubes were prepared containing a small amount of sodium azide, a substance that prevents bacterial growth and sample contamination. Subjects provided saliva samples in small volumes (3–5 ml) that were deposited in tubes. The samples were frozen for several months, then thawed and analysed using standard protocols. These well-established procedures involve multiple levels of error correction.

Testosterone levels vary in a predictable fashion throughout the day. The experiment was conducted in the early afternoon because the rate of change in the diurnal cycle is lowest during this period. Accordingly, subjects provided baseline samples at 14.00 on 3 non-experimental days. On the day of the ultimatum game, subjects reported at 13.00 and made their decisions at approximately 14.00.

## 3. Results

**Figure 1:**
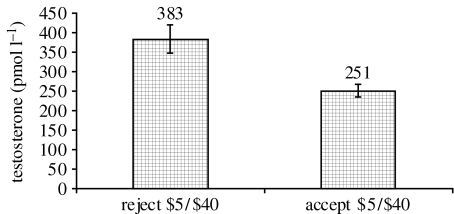
Subjects who reject $5 out of $40 have significantly higher testosterone levels than those who accept.

As predicted, men who rejected low ultimatum game offers ($5 out of $40) had significantly higher testosterone levels than those who accepted (*p*<0.01, one-sided *t*-test). For each subject, testosterone level is estimated by averaging three samples given on different days. As shown in table 1 and figure 1, rejecters (six) had an average testosterone level of 383 pmol l^−1^ versus an average of 251 pmol l^−1^ for those who accepted (20).

This result is robust over several tests. The effect is present beyond the *p*=0.05 value if logged values of testosterone are used instead of raw values. It is also present beyond the *p*=0.05 value if any one of the subjects is removed from the sample, or if any one of the subject's rejection behaviour is switched from reject to accept or vice versa.

Furthermore, the relationship between testosterone and behaviour is visible by looking at the data. For subjects with above-average testosterone, 45% rejected the $5 offer versus 7% of the men with below-average testosterone. Of the seven men with the highest testosterone levels, five rejected $5, whereas only one of the 19 men with the lowest testosterone levels rejected $5.

It is also possible to examine the relationship between ultimatum game offers and testosterone levels. Table 2 summarizes the finding that men who offered $25 out of $40 had higher average testosterone levels than those who offered $5 out of $40, but this difference is not significant at the *p*=0.05 level. Men who made larger offers (11) had an average testosterone level of 313 pmol l^−1^ versus an average of 257 pmol l^−1^ for those who made smaller offers (15), (*p*>0.10, two-sided *t*-test). The two-sided *t*-test was used because there was no pre-experimental hypothesis regarding ultimatum game offers and testosterone levels.

## 4. Discussion

‘Moralistic aggression’ is Robert Trivers' term for punishment used to modulate reciprocal altruism (Trivers 1971). In this study, punishment of low ultimatum game offers is correlated with high levels of testosterone. This finding was predicted based on a hypothesis that ultimatum game rejections are caused, at least in part, by psychological mechanisms for reciprocal altruism being mobilized in an experimental environment constructed to make reciprocity impossible. In settings where people might interact more than once, punishment may enhance the reputation of the punisher, and it may alter the behaviour of the punished. Both routes may produce benefits to the punisher that exceed the cost of punishment.

The result of this study is consistent with prior work reporting increased neural activation in response to low ultimatum game offers (Sanfey *et al.* 2003). An evolved psychology to modulate reciprocal altruism ought to be very concerned about unequal divisions. An interesting follow-up study would be to look at both testosterone levels and neural activation in the presence of low ultimatum game offers. The suggestion is that high-testosterone men would have relatively stronger emotional responses to low offers.

A number of further testosterone experiments are suggested. It would be useful to replicate this study with more individuals and subjects drawn from a variety of populations. There is also a literature on biological responses to challenges (Sapolsky 1990; Wagner *et al.* 2002) that suggests studies looking at hormonal changes after economic actions.

This study used only men because testosterone seems to play a more central role in male behaviour than in female behaviour. However, there is a considerable literature on women and testosterone that reveals some of the same correlations between testosterone and behaviour (Dabbs & Hargrove 1997; Grant & France 2001).

An obvious extension would be to study testosterone in other well-known economic games, such as the prisoners' dilemma, dictator game and public goods game. However, this study alone does not provide clear predictions for behaviour in other settings. If testosterone is a useful proxy for human status, then a prediction requires an understanding of the relationship between behaviour and status in the relevant context. While the literature on the response of dominants to challenges is deep and relatively unambiguous, the existence of similar literature relevant to other economic games is less apparent.

There are other well-known biological markers that might be relevant to the study of economic behaviour. Fluctuating asymmetry (FA), for example, is a frequently used measure of developmental stability and is correlated with a range of behaviours in myriad species (Gangestad & Thornhill 1999). A study of FA and the ultimatum game reports that more asymmetric men (high FA) make larger ultimatum game offers, but finds no relationship between FA and ultimatum game rejections (Zaatari & Trivers 2007).

Ultimatum game rejections have become important because mainstream economic theory fails to predict this behaviour. The divergence between actual human behaviour and that predicted by economic theory has played a central role in the rise of behavioural economics. If our understanding of ultimatum game rejections and other related phenomena can be improved through studies of hormones, morphology and neurological activity, the ramifications for economics might be quite broad and positive.

## Figures and Tables

**Table 1:** Testosterone level in men and their responses in an ultimatum game.

	*N*	average testosterone (pmol l^−1^)	s.e.
reject $5/$40	6	383	37
accept $5/$40	20	251	16
all	26	281	

**Table 2:** Testosterone level in men and their offers in an ultimatum game.

	*N*	average testosterone (pmol l^−1^)	s.e.
offer $25/$40	11	313	33
offer $5/$40	15	257	19
all	26	281	
